# More Than One HMG-CoA Lyase: The Classical Mitochondrial Enzyme Plus the Peroxisomal and the Cytosolic Ones

**DOI:** 10.3390/ijms20246124

**Published:** 2019-12-04

**Authors:** María Arnedo, Ana Latorre-Pellicer, Cristina Lucia-Campos, Marta Gil-Salvador, Rebeca Antoñanzas-Peréz, Paulino Gómez-Puertas, Gloria Bueno-Lozano, Beatriz Puisac, Juan Pié

**Affiliations:** 1 Unit of Clinical Genetics and Functional Genomics, Department of Pharmacology-Physiology, School of Medicine, University of Zaragoza, CIBERER-GCV02 and ISS-Aragon, E-50009 Zaragoza, Spain; marnedo@unizar.es (M.A.); alatorre@unizar.es (A.L.-P.); cristinaluca96@hotmail.com (C.L.-C.); martage.sc@gmail.com (M.G.-S.); rebecantop@gmail.com (R.A.-P.); 2 Molecular Modelling Group, Center for Molecular Biology “Severo Ochoa” (CSIC-UAM), Cantoblanco, E-28049 Madrid, Spain; pagomez@cbm.csic.es; 3 Department of Paediatrics, Hospital Clínico Universitario “Lozano Blesa”, E-50009 Zaragoza, Spain; mgbuenol@unizar.es

**Keywords:** HMG-CoA lyase, *HMGCL*, *HMGCLL1*, ketone bodies, 3-hydroxy-3-methylglutaric aciduria

## Abstract

There are three human enzymes with HMG-CoA lyase activity that are able to synthesize ketone bodies in different subcellular compartments. The mitochondrial HMG-CoA lyase was the first to be described, and catalyzes the cleavage of 3-hydroxy-3-methylglutaryl CoA to acetoacetate and acetyl-CoA, the common final step in ketogenesis and leucine catabolism. This protein is mainly expressed in the liver and its function is metabolic, since it produces ketone bodies as energetic fuels when glucose levels are low. Another isoform is encoded by the same gene for the mitochondrial HMG-CoA lyase (*HMGCL*), but it is located in peroxisomes. The last HMG-CoA lyase to be described is encoded by a different gene, *HMGCLL1,* and is located in the cytosolic side of the endoplasmic reticulum membrane. Some activity assays and tissue distribution of this enzyme have shown the brain and lung as key tissues for studying its function. Although the roles of the peroxisomal and cytosolic HMG-CoA lyases remain unknown, recent studies highlight the role of ketone bodies in metabolic remodeling, homeostasis, and signaling, providing new insights into the molecular and cellular function of these enzymes.

## 1. Introduction

3-Hydroxy-3-methylglutaryl coenzyme A (HMG-CoA) lyase (HL) catalyzes the cleavage of 3-hydroxy-3-methylglutaryl CoA into acetoacetate (AcAc) and acetyl-CoA. More than 60 years have elapsed since the discovery of this enzyme in pig heart [1]. After that, HL has been functionally and structurally well characterized in vertebrates [2], bacteria [3], and plants [4]. Both plants and vertebrates have three isoenzymes of HLs located in three different cellular compartments: mitochondria, cytosol, and peroxisomes [5,6,7,8]. Therefore, various regulation mechanisms for the synthesis of ketone bodies and/or amino acid catabolism could exist.

The mitochondrial HMG-CoA lyase (mHL) was the first human HL described and its role in mammalian metabolism has been extensively studied [9]. During ketogenesis, mHL catalyzes the transformation of HMG-CoA into acetoacetate, the first type of ketone body. Later, in the 90s, the existence of another enzyme with HMG-CoA lyase activity that could produce ketone bodies in the cell was reported. It is encoded by the same gene for mHL, *HMGCL*, but it is located in a different subcellular compartment, the peroxisomes [8]. There is less information available about the peroxisomal isoform of HL (pHL), and its function is still barely known. More recently, in 2004, genomic databases reported a new gene, namely, *HMGCLL1* with high homology to *HMGCL.* This gene encoded a novel isoform of HL (er-cHL), which also had lyase activity and was capable of synthesizing acetoacetate and acetyl-CoA [6,7]. Nevertheless, its subcellular location in the cytosol and endoplasmic reticulum and its activity and tissue distribution were different from mHL and pHL, suggesting a different function for this isoform [6,7].

The existence of three enzymes with HMG-CoA lyase activity and the fact that they are able to synthesize ketone bodies in different subcellular compartments is surprising. Why does the cell need the presence of acetoacetate or β-hydroxybutyrate (BHB) in the peroxisome or the cytosol? It seems that the several functions that ketone bodies play in the cells could be related to their location in different subcellular compartments. In this review, we compare for the first time, the three human isoforms of the HMG-CoA lyase from different scopes, such as metabolic, molecular biology, phylogenetic, and clinical scopes, focusing on their different features and describing their tentative roles.

## 2. Ketone Body Metabolism

Ketone body metabolism, including ketogenesis and ketolysis, is considered a central metabolic process during several physiological conditions, such as fasting, caloric restriction, low carbohydrate diets, high-intensive exercises, pregnancy, or neonatal periods [10]. The major ketone bodies are β-hydroxybutyrate, acetoacetate, and acetone. They are mainly synthesized in hepatic mitochondria through the ketogenesis pathway, serving as an energy carrier for extrahepatic tissues. In addition, two novel metabolic pathways for the synthesis of ketone bodies have been described in the cytosol and peroxisomes, suggesting different regulation mechanisms of synthesis, and other tentative roles beyond energy fuel [6,11]. Moreover, some crucial molecular processes are inter-related with ketone bodies, such as β-oxidation (FAO), the tricarboxylic acid cycle (TCA), or the biosynthesis of lipids, steroids, and amino acids. Furthermore, ketone body metabolism determines the balance of cellular metabolic pairs (NADH/NAD^+^ and AcAc/BHB), the cellular concentration of critical signaling metabolites (acetyl-CoA), and the cellular production of BHB, thus increasingly being recognized as a cellular second messenger [10].

Acetyl-CoA, the substrate for hepatic ketogenesis, derives mainly from FAO, and to a lesser extent, from the catabolism of amino acids, especially leucine. The initial ketogenesis step consists of a reversible reaction in which two molecules of acetyl-CoA form acetoacetyl-CoA and the reaction is catalyzed by acetoacetyl-CoA thiolase (ACAT1). A third acetyl-CoA molecule is then condensed to form 3-hydroxy-3-methylglytaryl-CoA (HMG-CoA) by the mitochondrial HMG-CoA synthethase (HMGCS2). After that, HMG-CoA is transformed into AcAc and acetyl-CoA by mHL. Then, the majority of acetoacetate is reduced to BHB by the mitochondrial β-hydroxybutyrate dehydrogenase (BDH1) in an NAD^+^/NADH coupled reaction. In some tissues, such as the lung, AcAc could be spontaneously decarboxylate into acetone.

**Figure 1:**
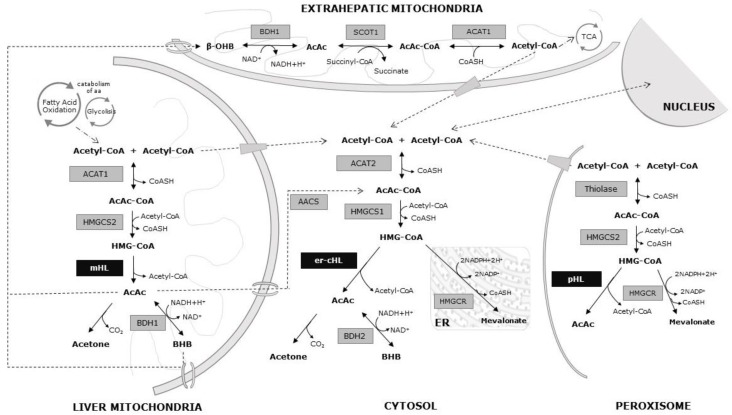
Metabolic pathways of the ketone bodies. Black arrows: chemical reactions at different cellular compartments. Dotted arrows: substrate transport pathways.

While acetone is not further catabolized to produce ATP, both AcAc and BHB are exported from liver mitochondria into the bloodstream and consumed by extrahepatic tissues. During ketolysis, BHB is converted back to two acetyl-CoA molecules by BDH1, 3-oxoacid CoA-transferase 1 (SCOT1), and ACAT1. These acetyl-CoAs feed into TCA to yield NADH for ATP synthesis via oxidative phosphorylation (Figure 1).

Additionally, acetyl-CoA and AcAc can be exported from the mitochondrial matrix to cytosol, being the precursors of multiple anabolic reactions related to fatty acid, steroid, and amino acid synthesis. An extramitochondrial variant of HMG-CoA synthetase (HMGS1) employs one acetyl-CoA molecule to convert AcAc-CoA into HMG-CoA in the cytosol. It is well known that HMG-CoA in the cytosol is metabolized by hydroxy-3-methyl-glutaryl-CoA reductase (HMGCR) within the mevalonate pathway. It must be stressed that er-cHL is also able to cleave HMG-CoA into acetyl-CoA and AcAc, supporting a novel pathway for AcAc and BHB production in animal tissues (Figure 1).

This metabolic pathway is also completed in the peroxisomes of cells [8]. The acetyl-CoA from the cytosol can get through the peroxisomal membrane, where the thiolase condenses it to form acetoacetyl-CoA (Figure 1).

These metabolic pathways suggest a finely tuned regulation of ketone bodies synthesis. Two mechanisms are particularly relevant in this regulation, the substrate availability and the expression and activity of the enzyme HMGCS2 [12]. Although significant progress has been made in deciphering how ketogenic rates are regulated, researchers tend to focus on HMGCS2, which is classically considered to be the rate-limiting enzyme of ketogenesis. Therefore, more information is not available about the direct regulation of HMG-CoA lyase enzymes affecting the final pool of ketone bodies. An alternative splicing mechanism has been proposed for the regulation of the active forms of *HMGCL* and *HMGCS2* genes in a tissue-specific manner [13]. Alternative inactive splicing isoforms from *HMGCL* and *HMGCS2* genes are expressed at high levels in tissues that do not produce ketone bodies, such as heart, skeletal muscle, and brain, whereas the expression of the complete active isoform is low in these tissues. On the other hand, the expression level pattern of these isoforms is the opposite in tissues that produce ketone bodies at high rates [13]. Moreover, recent studies show that mitochondrial and peroxisomal HLs may inactivate cytosolic HL due to specific physiological requirements in *Arabidopsis thaliana* [4].

## 3. Roles of Ketone Bodies: More Than Energy Fuel

It is widely known that the main role of ketone bodies is bioenergetics. In normal circumstances, the energy yield from mitochondrial oxidation of AcAc or BHB is around 22 ATP molecules. This metabolic pathway is particularly relevant for some tissues, such as brain, where ketolysis accounts for between 60% and 70% of the energy supply during glucose deprivation [14]. The use of ketone bodies as an alternative energy fuel by the brain is an example of the importance of these metabolites, because the brain is able to use glucose and ketone bodies only to obtain energy [14]. Moreover, ketone bodies are used as a substrate for the synthesis of lipids in the brain [15], so their role as lipid precursors is imperative in the growth and development of brain [16].

Emerging studies have recently shown the potential importance of ketone bodies not only in cell metabolism, but also in metabolic remodeling, homeostasis, and signaling [10].

### 3.1. Metabolic Remodeling

Apart from physiological conditions, ketone bodies are essential elements in cellular metabolic remodeling during diseases and disorders. This is true for heart, where several studies show that ketone utilization is increased in both animal models and human failing hearts [17,18], although there are still discrepancies as to whether this increase in BHB oxidation improves either cardiac work or efficiency in failing hearts [19,20]. In addition, utilization of ketones has been shown to be upregulated in diabetes mellitus [21] and an increase in AcAC utilization has been recently observed in the diabetic rat heart [21]. In brain, ketone bodies can act as neuroprotective molecules. In fact, it is widely believed that the antioxidant effects of BHB and AcAc play a vital role in the prevention of neurodegeneration. The capability of BHB to reduce the production of reactive oxygen species (ROS) and prevent neuronal death was demonstrated in several models [22,23], but its mode of action is unclear. Several hypotheses point that ketone bodies can activate glutathione peroxidase and decrease ROS production [24]. Others have shown that ketone bodies reduce the production of ROS by improving mitochondrial respiration and bypassing the complex I dysfunction [25]. The ketogenic diet has been studied as a treatment in patients with complex I deficiency, and although there are a few published studies, the results show a clinical improvement of the patients [26,27]. Besides that, it has long been known that BHB is able to activate the hydroxycarboxylic acid receptor 2 (HCA2) on adipocytes in order to reduce lipolysis [28]. Interestingly, HCA2 expression is not confined to adipocytes, but is also present on neutrophils, macrophages, and brain. Therefore, BHB is able to activate a neuroprotective subset of macrophages via HCA2 receptors [29]. In any case, based on these protective effects, the ketogenic diet is used in the treatment of several diseases, such as Parkinson’s disease and Alzheimer’s disease [30].

### 3.2. Physiological Homeostasis

There is clear evidence that ketone bodies play a major role in the maintenance of physiological homeostasis and it is believed that they alter pathways in the aging process [31]. Although the mechanism underlying the anti-aging effects of ketone bodies has not been fully elucidated, recent studies provided strong evidences supporting their anti-inflammatory [32] and anti-oxidative effects [33,34]. Thus, prevention of senescence by BHB has been demonstrated in vascular cells [35]. Ketone bodies can modulate inflammatory and immune cell function. Mechanistically, BHB is able to inhibit NLRP3 (NACHT, LRR and PYD domains-containing protein 3) inflammasomes [32]. Furthermore, a ketone body shuttle between hepatocytes and local macrophages has been demonstrated. It should be pointed out that AcAc and BHB have segregated roles, and that AcAc is the one that protects the liver against fibrosis [36]. Among the most recent discoveries, the stemness regulation in intestinal stem cells by the production of endogenous ketone bodies is remarkable [37]. 

### 3.3. Signaling

In the last years, several studies have been published supporting the idea that the ketone bodies, and more specifically, the BHB, are signaling metabolites. In this sense, BHB could modify proteins at a post-transcriptional level, in a reaction named β-hydroxybutyrylation (kbhb). This was initially described in histone proteins, and, since then, a total of 44 histone kbhb sites have been identified in both human and mouse cells, suggesting that a variety of biological events could be regulated by ketone bodies [38]. Besides histone post-transcriptional modifications, a BHB-mediated kbhb has been described in a transcriptional factor, p53, as a novel mechanism by which ketone bodies might be involved in gene expression regulation [39]. Furthermore, since ketone bodies contribute to compartmentalized pools of acetyl-CoA, they could be involved in histone lysine acetylation dynamics [40]. Additionally, ketone bodies could act as signaling metabolites by inhibiting the activity of class I histone deacetylases (HDACs) [33]. All these findings suggest that BHB could be a key transcription and epigenetic regulator. 

All these data suggest that ketone bodies and their metabolism are more important to cells than it was originally thought, and this fact could explain the existence of more than one HMG-CoA lyase enzyme in different subcellular compartments.

## 4. Gene Structure and Protein Features of HMG-CoA Lyases

**Figure 2:**
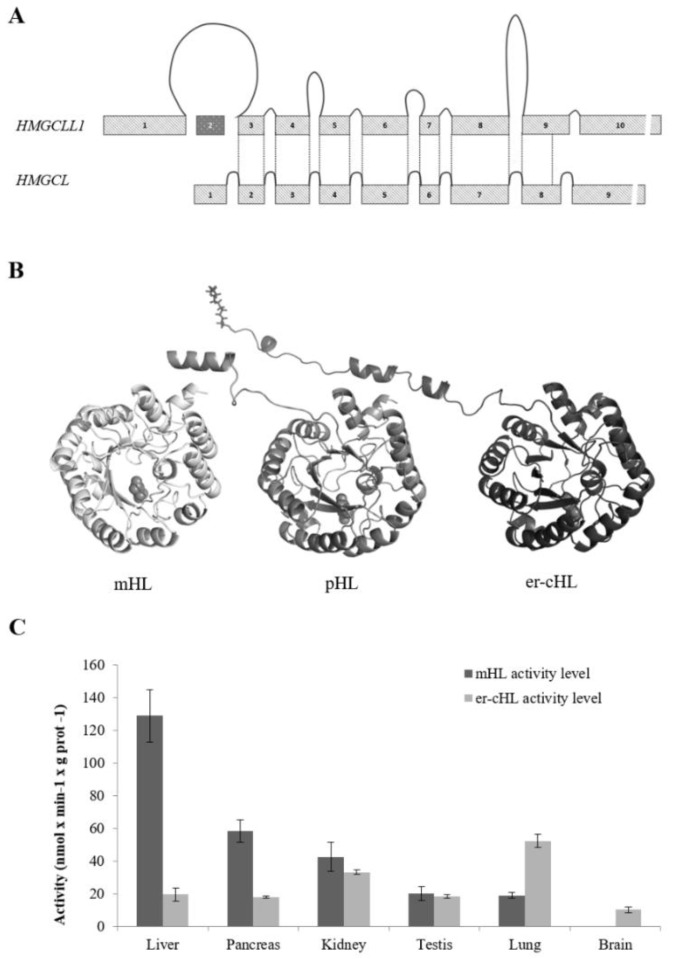
Gene and protein structure, and enzymatic activity comparison of mHL, pHL, and er-cHL isoenzymes. (**A**) *HMGCLL1* and *HMGCL* gene structures. The size of the exons and introns of both genes is represented in scale. The lines represent the regions that encode the equivalent protein structure in both genes. (**B**) 3D structure model for human mHL, pHL, and er-cHL. The three proteins share a TIM-barrel structure. The mHL protein cleaves its mitochondrial leader peptide, which is preserved in the pHL protein. The myristic acid is represented in the er-cHL protein structure at its amino-terminal end. (**C**) Comparison of activity levels of er-cHL and mHL proteins in different adult human tissues. This research was originally published in the *Journal of Lipid Research*. Arnedo M. et al. Characterization of a Novel HMG-CoA Lyase Enzyme with a Dual Location in Endoplasmic Reticulum and Cytosol. *J. Lipid Res.* 53 (10), 2046–2056 [6].

The mHL, pHL, and er-cHL lyases show properties of isoenzymes encoded by two distinct functional genes. Whereas mitochondrial and peroxisomal enzymes are encoded by the *HMGCL* gene (NT_004610.17), located at chromosome 1p36.1–p35, er-cHL is encoded by the *HMGCLL1* gene (NT_007592.15) at chromosome 6p12.1. These genes show a high degree of sequence homology and similar exon/intron structure (Figure 2A). While both the genes have an open reading frame of about 1000 nt in length, the gene sizes are extremely different: *HMGCL* consists of 24 kb and *HMGCLL1* consists of 145 kb. This variance in intron length could indicate differences in gene expression regulation [41,42]. It has been suggested that intron-poor structures are commonly found in genes involved in rapid biological responses [41]. In contrast, intron size is highly conserved in genes associated with embryonic development, since transcriptional delay is functionally significant [42].

At least two physiological splicing variants have been reported for the *HMGCL* gene [13]—one bearing a deletion of exons 5 and 6 (*HMGCLΔ5,6*) and the other with a deletion of exons 5, 6, and 7 (*HMGCLΔ5,6,7*)—both lacking enzymatic activity. On the other hand, six splicing isoforms have been described in databases and experimentally by Arnedo et al. (2012) for the *HMGCLL1* gene, although their possible function, in the tissues where they were found, remains unstudied.

Nevertheless, the main *HMGCLL1* isoform (NM_001042406.1) encodes a protein with a structure very similar to mHL and pHL (Figure 2B). All of them have a TIM-barrel structure with an auxiliary domain composed of a β-sheet and two α-helices [2,43]. A homodimer structure was confirmed in the three isoenzymes, even in pHL [44], which had been previously described as a monomer [11]. The interaction between the monomers is located at the β9 sheet and the α11 and α12 helices, which are not components of the barrel.

The deduced amino acid sequences of mHL and er-cHL are 83% identical. The main feature of HL isoenzymes is the HMGL-like domain responsible for their lyase activity, which is located between amino acids 33 and 306 in mHL and 78 and 345 in er-cHL. The most critical amino acids for the catalytic activity of the enzymes (Arg^41^, Glu^72^, Tyr^167^, and Cys^266^ in mHL and Arg^56^, Glu^87^, Tyr^182^, and Cys^281^ in er-cHL) as well as the residues that bind to the divalent cation Mg^+2^, which is essential for the lyase activity (Asp^42^, Asn^275^, His^233^, and His^235^ in mHL and Asp^57^, Asn^288^, His^248^, and His^250^ in er-cHL), are well known. Furthermore, their location has been the same in the mHL secondary structure elements along evolution [45].

The three isoenzymes of HL can be distinguished by their N-terminal sequence. The mHL enzyme contains a mitochondrial leader peptide that extends from the first to the 27th amino acid. In the pHL enzyme, the C-terminal tripeptide (CKL) leads the protein to peroxisomes. The er-cHL protein has an N-myristoylation motif (MGNVPSA) at its N-terminal sequence that is likely related to its association with the cellular membranes [7]. Besides that, subcellular localization of HLs has been experimentally demonstrated. Several assays of subcellular activity [5] and fluorescence co-localization studies [6] confirmed the mitochondrial subcellular location for mHL. Peroxisomal localization of pHL was established by subcellular fractionation and enzymatic activity measures in samples obtained from mouse liver [8]. Whereas er-cHL localization was confirmed in the cytosolic side of the endoplasmic reticulum membrane, the interaction between the protein and the membrane is weak and the enzyme can be found dissociated as well [6].

Additionally, the three proteins were expressed in a bacterial model in order to study their lyase enzymatic activity. Although there were variations among the different assays, lyase enzymatic activity was confirmed in mHL, pHL, and er-cHL [6,7,45,46,47,48,49].

Finally, an HL enzyme has been described in *Arabidopsis thaliana*, where the *At2g26800* gene is able to encode three different isoforms of the HMG-CoA lyase [4]. The alignment of the diverse isoforms with human mHL showed a high similarity, where all the key amino acids for coordination with Mg^+2^ or interaction with the CoA ligand are conserved. The structural model of the different HLs of *A. thaliana* also revealed a great homology with the human lyases [4].

## 5. Phylogenetic Evolution of the HMG-CoA Lyases

The strong structural similarity of mHL and er-cHL suggests that the genes encoding them have a common origin. By using MUSCLE [50] to perform a multiple sequence alignment and refining the alignment using GBLOCKS [51], a maximum likelihood phylogenetic tree can be built [52,53] which helps us to understand the phylogenetic evolution of these enzymes.

As commented above, the HLs have a TIM-barrel structure, which is common in enzymes that catalyze metabolic reactions in the body. In fact, they belong phylogenetically to one of the oldest protein families: the beta/alpha barrel scaffold. Initially, this family of proteins was arranged in the form of a hemibarrel. The fusion of two structurally equivalent genes would result in the appearance of the complete barrel. From here, the next evolutionary steps would take place by gene duplication. Each time this occurred, two new enzymes would emerge, which over time would specialize in different functions [54].

**Figure 3:**
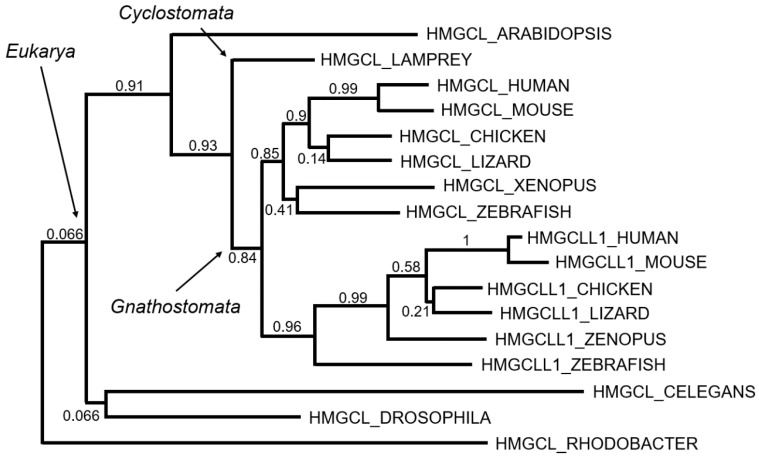
Tentative evolution of the HMG-CoA lyases. Maximum likelihood phylogenetic tree based on multiple sequence alignment is shown in Figure S1. Bootstrap values (0, lowest to 1, highest) are indicated as measurements of branch confidence scores.

Following this hypothesis, it was plausible to speculate that *HMGCL* and *HMGCLL1* appeared by gene duplication. The phylogenetic comparison showed that the oldest gene was *HMGCL* (Figure 3). Enzymes of analogous structures have been found in bacteria, specifically Alphaproteobacteria, such as *Rhodobacter*, *Rhizobium*, *Sphingomonas*, *Rhodospirillum,* and *Caulobacter*. It is likely that the symbiont bacteria of eukaryotes carried the *HMGCL* gene. The absence of this gene in mitochondrial DNA would be explained by the usual migration of this genetic material to the nuclear DNA of eukaryotic cells. 

Assuming the idea that the first enzyme was the mitochondrial lyase, we could wonder when the gene duplication that led to the appearance of the *HMGCLL1* gene occurred. Sequence homology suggests that *HMGCLL1* appeared for the first time in vertebrates with mandibles, Gnathostomata, about 420 million years ago (Figure 3). In this period, there was a division between Agnatha (lamprey and related) and Gnathostomata by complete genomic duplication [55,56]. Humans come from this last branch and it is believed that this was the origin of many of the genes that appeared duplicated in the human genome.

Moreover, a convergent-like evolution has been recently described in plants. The HL isoforms have been found at the same localizations (mitochondria, peroxisome, and cytosol) as higher organisms. The subcellular location in these three cell compartments can be explained for the alternative splicing of the *HMGCL* gene, as it has been described in the case of *Arabidopsis thaliana* [4].

## 6. Tissue-Specific Expression of HMG-CoA Lyases

Although mHL function has been deeply studied, relatively very little is known about er-cHL and pHL. The different tissue distribution of the three human lyase enzymes could provide insights into their specific function. 

The widespread adoption of RNA-seq and the completion of large projects, such as ENCODE [57] and GTEx [58], give us a valuable opportunity to study the tissue-specific expression of *HMGCL* and *HMGCLL1*. As it would be expected, the liver is the tissue that shows the highest mRNA expression level of *HMGCL*, since it is the main ketogenic tissue. Interestingly, *HMGCLL1* is preferentially expressed in brain and lung tissues [59]. Furthermore, the analysis of *HMGCLL1* expression using the web-based application http://evodevoapp.kaessmannlab.org [58] reinforced these data and suggested an important role of *HMGCLL1* during fetal brain development.

Besides public datasets, tissue-specific distribution of mHL and er-cHL has been confirmed not only at the mRNA level, but at the protein and enzymatic activity levels as well. Thus, the liver is the tissue that shows the highest mHL activity, as well as the highest protein and mRNA expression levels [5]. A high activity level was also found in pancreas and kidney. Interestingly, mHL activity was not detected in the brain mitochondrial fraction, but a significant activity of er-cHL was reported in the brain, despite the difficulties in studying the human brain immediately after death due to the fast degradation of proteins. The highest er-cHL activity was found in lung tissues [6] (Figure 2C). Overall, gene expression data found in public databases were experimentally confirmed. Unfortunately, there is very less information about the tissue distribution of the pHL isoform and its function in the peroxisomes is yet to be extensively studied.

Together, these observations underscore the importance of tissue-specific expression of mHL and er-cHL, possibly reflecting a response to different signals as well as different roles in the synthesis of ketone bodies (see Section 3, above).

## 7. HMG-CoA Lyase Related Diseases

To date, only the deficiency of the mHL protein has been reported to cause a genetic disease, 3-hydroxy-3-methylglutaric aciduria (OMIM 246450) [60]. The absence of this enzyme causes ketone body production failure and the accumulation of toxic acid metabolites of leucine catabolism that may disrupt normal organ function. For this reason, it is included within alterations of fatty acid metabolism and within organic acidurias.

Since the description of the deficiency in 1976, more than 150 patients have been reported in the literature [47,61]. It happens in all ethnic groups throughout the world, but with a greater incidence in countries of the Mediterranean area, Spain and Portugal [47] and in Saudi Arabia [62]. It is considered a rare disease of autosomal recessive nature with a very low incidence (<1/100,000 among live newborns). Despite its low frequency, it is recommended that hospitals with neonate units should have the capacity to diagnose it early, because its treatment is effective and simple [63].

Although there is a considerable clinical heterogeneity, in most cases, the disease appears in the first year of life, and in about 50% of patients, the disease appears in the neonatal period, which highlights the importance of ketone bodies in the brain metabolism of newborns. In rare cases, it can also be found in adolescents or adults [64,65,66].

The onset of symptoms is initiated by fasting, infection, dietary protein load, or the stress of birth. Affected patients commonly present acute clinical manifestations, such as metabolic acidosis and hypoketotic hypoglycemia, accompanied by vomiting, dehydration, hypotonia, and lethargy. Other chronic manifestations of the disease are less frequent and include hepatomegaly, macrocephaly, and less frequently microcephaly, seizures, dilated cardiomyopathy, arrhythmia, and acute pancreatitis. As already discussed, the finding of higher enzymatic activity in pancreas [5] justifies that this organ may be injured by the accumulation of toxic metabolites. 

Abnormalities in cerebral white matter and basal ganglia have also been reported, although the pathogenesis of brain damage in this disease is poorly known. As in pancreas, it has been proposed that the acid compounds accumulating in 3-hydroxy-3-methylglutaric aciduria induce a pro-inflammatory response in astrocytes that may possibly be involved in the neuropathology of this disease [67].

This deficiency is a life-threatening condition and may cause irreversible coma and/or death; however, prognosis is good for the patients and the symptoms often improve around puberty. While the overall physical development of the patients appeared normal, the cognitive outcome was highly variable [61], perhaps due to vulnerability and affectation of the brain. Besides, pregnancy is considered especially stressful due to constant changes in fetal growth and subsequent metabolic demand. Several cases of metabolic acidosis and hypoglycemia during pregnancy, delivery, and postpartum period have been described that can be fatal if not treated appropriately [68].

Patients with HMG-CoA lyase deficiency show a diagnostic urinary organic acid pattern, with high levels of 3-hydroxy-3-methylglutaric acid, 3-hydroxy-isovaleric acid, 3-methylglutaric acid, and 3-methylglutaconic acid. A confirmation diagnosis requires direct enzyme activity testing or a genetic study of mutations in the gene *HMGCL*. So far, several mutations have been reported in this gene to cause a decrease in the HMG-CoA lyase activity [61,69,70,71,72,73]. At present, despite the considerable progress made in the genetic diagnosis of the deficiency, thanks to the use of next-generation sequencing gene panels, genotype–phenotype correlations are difficult to establish.

Although no disease has been reported to be related to pHL or er-cHL deficiency, a decrease in *HMGCLL1* gene expression level has been found in 18 cancer types [74]. Moreover, using the gene expression profiling interactive analysis (GEPIA) [75] that interrogated the TCGA pan-cancer and GTEx data repositories, a significant decrease in *HMGLL1* expression was observed in many cancer types, such as glioblastoma, lung adenocarcinoma, and testicular tumors. In contrast, *HMGCL* expression appears to be upregulated in several cancer tissues compared to corresponding normal tissues. This opposite behavior is highly surprising and further studies are required to characterize the roles of *HMGCL* and *HMGCLL1* expression in cancer cells. Connections between ketone bodies and cancer are under active investigation, and beyond the realm of fuel metabolism, the latest studies highlight the imperative roles of ketone bodies in tumor cell signaling [39,76,77].

Recently, a study reported that a specific *HMGCLL1* haplotype acts as a genetic biomarker for intrinsic sensitivity to tyrosine kinase inhibitor therapy in chronic myeloid leukemia (CML) patients [78]. The study suggested that inhibition of the *HMGCLL1* gene could be a potential therapeutic strategy to improve the response to imatinib therapy in CML patients [78].

Apart from its possible implication in cancer, a mutation in the *HMGLL1* gene has been reported in a patient with hypopituitarism [79], although its relevance in this disease has not been deeply studied given the fact that the patient also carried another mutation in the *HESX1* gene, which has been thoroughly studied in patients with hypopituitarism.

## 8. Conclusions

For many years, ketone body metabolism has been mainly associated with energy fuel and lipid metabolism. During the last 10 years, an increasing burst of studies on the functions of ketone bodies have been published. These studies provide insights into the key role of ketone bodies in metabolic remodeling, signaling, and homeostasis. Therefore, it has been proposed that ketone body synthesis pathways in cytosol and peroxisomes are crucial issues with attractive conceptual potential that requires a more thorough research. In this context, HMG-CoA lyase isoenzymes could be exciting tools to clarify the potential importance of ketone body synthesis in cytosol or peroxisomes, and in poorly explored tissues, such as the brain or lung. Addressing these questions promises to offer an exciting task for the years to come.

## Supplementary Materials

The following are available online at https://www.mdpi.com/1422-0067/20/24/6124/s1, Figure S1: Multiple sequence alignment of representative sequences from the HMGCL (mHL) and HMGCLL1 (er-cHL) family of proteins. Sequences were obtained from Uniprot (www.uniprot.org) and correspond to organisms: *Human, Mouse, Chicken, Anole lizard, Xenopus, Zebra fish, Sea lamprey, Drosophila, C. elegans, Arabidopsis and Rhodobacter*.

## Abbreviations

HMG-CoA	3-Hydroxy-3-methylglutaryl coenzyme A
HL	HMG-CoA lyase
mHL	Mitochondrial HMG-CoA lyase
pHL	Peroxisomal HMG-CoA lyase
er-cHL	HMG-CoA lyase-like 1
BHB	β-Hydroxybutyrate
AcAc	Acetoacetate
FAO	β-Oxidation
TCA	Tricarboxylic acid cycle
ACAT1	Acetoacetyl-CoA thiolase
HMGCS1	HMG-CoA synthetase 1
HMGCS2	HMG-CoA synthetase 2
SCOT1	3-Oxoacid-CoA 1
BDH1	β-Hydroxybutyrate dehydrogenase
HMGCR	HMG-CoA reductase
ROS	Reactive oxygen species
HCA2	Hydroxycarboxylic acid receptor 2
Kbhb	β-Hydroxybutyrylation
HDAC	Class I histone deacetylase
CML	Chronic myeloid leukemia
