# Supplementary material for: More Than One HMG-CoA Lyase: The Classical Mitochondrial Enzyme Plus the Peroxisomal and the Cytosolic Ones
Source: Int J Mol Sci. 2019 Dec 4;20(24):6124. doi: 10.3390/ijms20246124 (PMC6941031; doi:10.3390/ijms20246124)
Supplement: Supplementary file 1 [file ijms-20-06124-s001.pdf]

```
HMGCL_CELEGANS      1 -----------------------------------------M------------------
HMGCL_RHODOBACT     1 ------------------------------------------------------------
HMGCL_DROSOPHIL     1 -----------------------------------------MIS----------------
HMGCL_ARABIDOPS     1 MQWNGVRRAHSIWCKRLTNNTHLHHPSIPVSHFFTMSSLEEPLSFDKLPSMSTMDRIQRF
HMGCLL1_ZEBRAFI     1 -------------------------------MGNVSSAVKHCLSYETF------------
HMGCLL1_XENOPUS     1 -------------------------------MGNVPYAVKQCISYQHL------------
HMGCLL1_HUMAN       1 -------------------------------MGNVPSAVKHCLSYQQL------------
HMGCLL1_MOUSE       1 -------------------------------MGNLPSAAKHCLNYQQL------------
HMGCLL1_CHICKEN     1 -------------------------------MGTVPSALKHCLSYQHL------------
HMGCLL1_LIZARD      1 -------------------------------MGNVPSALKHCLSYQHL------------
HMGCL_XENOPUS       1 ------------------------------------------MSFRRL------------
HMGCL_ZEBRAFISH     1 ----------------------------------------MAALMMRV------------
HMGCL_LAMPREY       1 ------------------------------------------------------------
HMGCL_HUMAN         1 -----------------------------------------MAAMRKA------------
HMGCL_MOUSE         1 -----------------------------------------MASVRKA------------
HMGCL_CHICKEN       1 -----------------------------------------MAAVRRL------------
HMGCL_LIZARD        1 -----------------------------------------MVVANRA------------

HMGCL_CELEGANS      2 ------------LPNRLL------------------------------------------
HMGCL_RHODOBACT     1 ------------MP----------------------------------------------
HMGCL_DROSOPHIL     4 ------------APIRSIL-----------------------------------------
HMGCL_ARABIDOPS    61 SSGACRPRDDVGMGHRWIEGRDCTTSNSCIDDDKSFAKESFPWRRHTRKLSEGEHMFRNI
HMGCLL1_ZEBRAFI    18 ------------LRDYPWL-----------------------------------------
HMGCLL1_XENOPUS    18 ------------LKDQFWNA----------------------------------------
HMGCLL1_HUMAN      18 ------------LREHLWIGD---------------------------------------
HMGCLL1_MOUSE      18 ------------LREHLWSGD---------------------------------------
HMGCLL1_CHICKEN    18 ------------LKEQLWIG----------------------------------------
HMGCLL1_LIZARD     18 ------------LKEPLWVGD---------------------------------------
HMGCL_XENOPUS       7 ------------VPVGWGAGW---------------------------------------
HMGCL_ZEBRAFISH     9 ------------AP-GPFAS----------------------------------------
HMGCL_LAMPREY       1 ------------------------------------------------------------
HMGCL_HUMAN         8 ------------LP-RRLVG----------------------------------------
HMGCL_MOUSE         8 ------------FP-RRLVG----------------------------------------
HMGCL_CHICKEN       8 ------------LP-RWAV-----------------------------------------
HMGCL_LIZARD        8 ------------IP-RLVA-----------------------------------------

HMGCL_CELEGANS      8 -----------------------------------------------ANRAYSTAINRFR
HMGCL_RHODOBACT     3 -----------------------------------------------------SFPPAVK
HMGCL_DROSOPHIL    11 -----------ALTA--------------------------------KRTAVTSAANQVR
HMGCL_ARABIDOPS   121 SFAGRTSTVSGTLRESKSFKEQKYSTFSNENGTSHI-----------SNKISKGIPKFVK
HMGCLL1_ZEBRAFI    25 ---------PRLLWEE-------------------------------KCSELPKLPVYVK
HMGCLL1_XENOPUS    26 ---------ETTLQE--------------------------------QESPPIGLPPHVK
HMGCLL1_HUMAN      27 -------SVAGALDPAQTSLLTNLHCFQPDVSGFSVSLAGTVACIHWETSQLSGLPEFVK
HMGCLL1_MOUSE      27 -------SVAGALDAAQ------------------------------EASQLPGLPEYVK
HMGCLL1_CHICKEN    26 -------EPTAPPHPG-------------------------------QESQASGLPEYVK
HMGCLL1_LIZARD     27 -------APPPPPSPPPTEDPQPLG----------------------QELLTSGFPEFVK
HMGCL_XENOPUS      16 -------TPLAILRQ--------------I-----------------GSLPIQSFPKEVK
HMGCL_ZEBRAFISH    16 ---------VRVNRPVLHCAASALIRSVSV-----------------SAAASQALPERVK
HMGCL_LAMPREY       1 ---------LSVLFQ--------------------------------GAVAISGLPNRVK
HMGCL_HUMAN        15 ---------LASLRA--------------V-----------------STSSMGTLPKRVK
HMGCL_MOUSE        15 ---------LTSLRA--------------V-----------------STSSMGTLPKQVK
HMGCL_CHICKEN      14 -----------SLRP---------------------------------VSTAAFPQRVK
HMGCL_LIZARD       14 -----------SLRP--------------L-----------------STAAATSLPKHVK

HMGCL_CELEGANS     21 VVEVGARDGLQAEKKFVPTEIKVELIDRLSECGFQTVETTSFVSPKWVPQLADHNEIVKK
HMGCL_RHODOBACT    10 IVEVGPRDGLQNEPRDIPVAERIALIEGLADAGLRVIEAGSFVSPRWVPQMAGTTEVLAG
HMGCL_DROSOPHIL    28 IVEVGPRDGLQNEPKLLPAATKIELINQLSETGLRTIEATSFVSAKWVPQMGDNAEVLKG
HMGCL_ARABIDOPS   170 IVEVGPRDGLQNEKNIVPTSVKVELIQRLVSSGLPVVEATSFVSPKWVPQLADAKDVMDA
HMGCLL1_ZEBRAFI    45 IVEVGPRDGLQNEKEIVPTEVKIQLIDLLSQTGLPVIEATSFVSSKWVAQMADHTAVLKG
HMGCLL1_XENOPUS    45 IVEVGPRDGLQNEKEFVPTDVKIEFIDQLSDTGLPAIEVTSFVSSKWVPQLSDHTEVMQG
HMGCLL1_HUMAN      80 IVEVGPRDGLQNEKVIVPTDIKIEFINRLSQTGLSVIEVTSFVSSRWVPQMADHTEVMKG
HMGCLL1_MOUSE      50 IVEVGPRDGLQNEKVIVPTDIKIELINQLSQTGLSVIEVTSFVSSRWVPQMADHAEVMRG
HMGCLL1_CHICKEN    48 IVEVGPRDGLQNEKVIVPTDTKIELINRLSKTGLPAIEVTSFVSSKWVPQMADHKEVMRG
```

```
HMGCLL1_LIZARD    58 IVEVGPRDGLQNEKAIVPTDVKIEFINRLSKTGLPVIEVTSFVSSKWVPQMADHTEVMGR
HMGCL_XENOPUS     38 IVEVGPRDGLQNEKTVVPTDVKIHLINMLSEAGLQAIEATSFVSPKWVPQMADHKNVMQG
HMGCL_ZEBRAFISH   50 IVEVGPRDGLQNEKTIVPTEVKIRLIDMLSEAGLPVIEATSFVSPKWVPQMADQEEVMRG
HMGCL_LAMPREY     20 IVEVGPRDGLQNEKTVVPTAVKIELIERLAEAGCPVVEATSFVSPKWVPQMADQVEVMAG
HMGCL_HUMAN       35 IVEVGPRDGLQNEKNIVSTPVKIKLIDMLSEAGLSVIETTSFVSPKWVPQMGDHTEVLKG
HMGCL_MOUSE       35 IVEVGPRDGLQNEKSIVPTPVKIRLIDMLSEAGLPVIEATSFVSPKWVPQMADHSDVLKG
HMGCL_CHICKEN     29 VVEVGPRDGLQNEKSVVPTPVKIRLIDMLSETGLPVIEATSFVSPRWVPQMADHAEVMQG
HMGCL_LIZARD      32 IVEVGPRDGLQNEKNIVPTQVKIDFINRLSETGLSVIEATSFVSPKWVPQMADHAEVMQG

HMGCL_CELEGANS    81 HRRFEGVSYPVLVPNAAGLKNALATGVVEEIAVFGAASDAFSLKNVNSNVEDSLKKLMEV
HMGCL_RHODOBACT   70 LRRDAGVSYPVLVPNMKGLDAAARAEGVEEIAVFAAASEAFSMRNINCSITESLARFSPV
HMGCL_DROSOPHIL   88 IRKVTGISYPVLTPNLKGFESALEA-GAEEVAVFGAASDAFSLKNVNCTAAEAIERFKPV
HMGCL_ARABIDOPS  230 VNTLDGARLPVLTPNLKGFQAAVSA-GAKEVAIFASASESFSLSNINCTIEESLLRYRVV
HMGCLL1_ZEBRAFI  105 IKRSPDVRYPVLTPNIQGFQAAVAA-GANEVAVFGSASETFSRKNINCSIEESLQRFEQV
HMGCLL1_XENOPUS  105 IKKHPGVRYPVLTPNIRGFHSAIAA-GATEVSVFGAASETFSRMNINCSIEESMTRFEDI
HMGCLL1_HUMAN    140 IHQYPGVRYPVLTPNLQGFHHAVAA-GATEISVFGAASESFSKKNINCSIEESMGKFEEV
HMGCLL1_MOUSE    110 IRQYPGVRYPVLTPNLQGFQHAVAA-GATEIAVFGAASESFSKKNINCSIEESMGRFQEV
HMGCLL1_CHICKEN  108 IERHPGVQYPVLTPNLKGFHSAIAA-GATEVSVFGAASESFSKMNINCSIEESIEKFEEV
HMGCLL1_LIZARD   118 IQRYPGVQYPVLTPNLHGFHSAIAA-GANEVSVFGAASEAFSKKNINCSIEESMERFENV
HMGCL_XENOPUS     98 IKKYPNISYPVLTPNLTGFQAAVEC-GAKEVAIFGAASELFSKKNINCSIDESLQRFKAV
HMGCL_ZEBRAFISH  110 LHKKPGVNYPVLTPNLKGFQAAVKA-GAKEVAIFGAASELFSKKNINCSVEESLVRFEEV
HMGCL_LAMPREY     80 IRKLPGVSYPVLTPNLKGFQAAVKA-GATEVAIFGAASESFSQKNINCSIAESLQRFEEV
HMGCL_HUMAN       95 IQKFPGINYPVLTPNLKGFEAAVAA-GAKEVVIFGAASELFTKKNINCSIEESFQRFDAI
HMGCL_MOUSE       95 IQKFPGINYPVLTPNMKGFEEAVAA-GAKEVSVFGAVSELFTRKNANCSIEESFQRFAGV
HMGCL_CHICKEN     89 INKLPGVSYPVLTPNLKGFQAAVAA-GAKEVSIFGAASELFTKKNINCSIEESLERFSEV
HMGCL_LIZARD      92 IQKVPGISYPVLTPNLKGFQAAVAA-GAKEVSIFGAASELFTKKNINCSIDESLERFSDV

HMGCL_CELEGANS   141 TKIALENNIRVRGYVSVVVGCPYQGAVQPEMVARVAEKLLESGCYEVSLGDTIGVGTVKT
HMGCL_RHODOBACT  130 VAAARARGWRVRGYVSCVLGCPYEGEVDPARVAEVAAELAALGCYEISLGDTIGVGTPLQ
HMGCL_DROSOPHIL  147 LKAAQKHGVRVRGYVSTVVGCPYEGAVAPSAVVKVVEALYQMGCYEISLGDTIGVGTPGT
HMGCL_ARABIDOPS  289 ATAAKEHSVPVRGYVSCVVGCPVEGPVLPSKVAYVVKELYDMGCFEISLGDTIGIGTPGS
HMGCLL1_ZEBRAFI  164 VSAAKQEGIPVRGYVSCALGCPYEGQVKPSQVTKVAKRLFELGCYEVSLGDTIGVGTAGS
HMGCLL1_XENOPUS  164 IKAAGNLNVPVRGYVSCALGCPYEGNIATSKVSEVSKRLYSMGCYEVSLGDTIGVGTPGS
HMGCLL1_HUMAN    199 VKSARHMNIPARGYVSCALGCPYEGSITPQKVTEVSKRLYGMGCYEISLGDTIGVGTPGS
HMGCLL1_MOUSE    169 ISSARHMDIPVRGYVSCALGCPYEGSITPQKVTEVSKRLYGMGCYEISLGDTIGVGTPGS
HMGCLL1_CHICKEN  167 AKSARNMNIPVRGYVSCALGCPYEGDITPAKVAEVSKRLYSMGCYEISLGDTIGVGTPGS
HMGCLL1_LIZARD   177 VKSARNMDIPVRGYVSCALGCPYEGSIAPAKVAQVSKRLYSMGCYEISLGDTIGVGTPGS
HMGCL_XENOPUS    157 ITEAKEANIPVRGYVSCVLGCPYEGKVAPSKVAEVAYKMFSMGCYEISLGDTIGVGTPGG
HMGCL_ZEBRAFISH  169 MTAAKQEGVSVRGYVSCVLGCPYEGKVSPSKVAEVAKRLYSMGCYEVSLGDTIGVGTPGG
HMGCL_LAMPREY    139 ARAAQDANIPVRGYVSCVVGCPYEGKINPAKVAEVAKCLYGMGCYEISLGDTIGVGTPGN
HMGCL_HUMAN      154 LKAAQSANISVRGYVSCALGCPYEGKISPAKVAEVTKKFYSMGCYEISLGDTIGVGTPGI
HMGCL_MOUSE      154 MQAAQAASISVRGYVSCALGCPYEGKVSPAKVAEVAKKLYSMGCYEISLGDTIGVGTPGL
HMGCL_CHICKEN    148 MNAARAASIPVRGYVSCVLGCPYEGNISAAKVAEVSKKMYSMGCYEISLGDTIGIGTPGS
HMGCL_LIZARD     151 LRAAKEANIPVRGYVSCVLGCPYEGKIAPAKVAEVSKKMYAMGCYEISLGDTIGVGTPGN

HMGCL_CELEGANS   201 VSKMLDTVLKSVPAEKLAVHFHDTYGQALANVLISIEKGIRSADSSIAGLGGCPYAKGAT
HMGCL_RHODOBACT  190 ARAMVGAVARQVPADRLAVHFHDTRGQALANVLAVLDQGIATVDASVAGLGGCPYAAGAS
HMGCL_DROSOPHIL  207 MRRMLDEVTKVVPAKDLAVHCHDTYGQALSNILVSLDYGIRVVDSSVSGLGGCPYAKGAS
HMGCL_ARABIDOPS  349 VVPMLEAVMAVVPADKLAVHFHDTYGQALANILVSLQMGISIVDSSIAGLGGCPYAKGAS
HMGCLL1_ZEBRAFI  224 MAEMLSDVLTEVPAGALAVHCHDTYGQALPNILIALQMGVSVVDASVAGLGGCPFAKGAS
HMGCLL1_XENOPUS  224 MRKMLESVMKEVPVSALAVHCHDTYGQALANILTAIQMGVSVVDCSVAGLGGCPYAKGAT
HMGCLL1_HUMAN    259 MKRMLESVMKEIPPGALAVHCHDTYGQALANILTALQMGINVVDSAVSGLGGCPYAKGAS
HMGCLL1_MOUSE    229 MKMMLESVMKEIPPGALAVHCHDTYGQALANILTALQMGINVVDSAVSGLGGCPYAKGAS
HMGCLL1_CHICKEN  227 MKRMLEAVMKEIPLSALAVHCHDTYGQALANILTAIQMGVAVVDSSVAGLGGCPYAKGAT
HMGCLL1_LIZARD   237 MRRMLESVMKEIPVTAIAVHCHDTYGQALANILTALQMGVHVVSLQCC-LGGCPYAKGAT
HMGCL_XENOPUS    217 MRDMLSAVLDVVPAKALAVHCHDTYGQALANILVALQMGVQVVDASVAGLGGCPYAQGAS
HMGCL_ZEBRAFISH  229 MTEMLNAVKKELPVEALAVHCHDTYGQALANILVALQNGVSVVDSSVAGLGGCPYAQGAS
HMGCL_LAMPREY    199 MREMLRAVSHEVPISALAVHCHDTYGQALANILTALQMGVSVVDSSVAGLGGCPYARGAS
HMGCL_HUMAN      214 MKDMLSAVMQEVPLAALAVHCHDTYGQALANTLMALQMGVSVVDSSVAGLGGCPYAQGAS
HMGCL_MOUSE      214 MKDMLTAVMHEVPVTALAVHCHDTYGQALANTLVALQMGVSVVDSSVAGLGGCPYAKGAS
HMGCL_CHICKEN    208 MKEMLAAVMKEVPVGALAVHCHDTYGQALANILVALQMGVSVVDASVAGLGGCPYAQGAS
HMGCL_LIZARD     211 MREMLSAVMKEVPIGALAVHCHDTYGQALANTLVALQMGVSVVDSSVAGLGGCPYAQGAS
```

```
HMGCL_CELEGANS   261 GNLATEDLLYFLKGNGFETGVNLDKVVETAKWFNEAAGYDKWSRVGAAMMAKKSKSCC--
HMGCL_RHODOBACT  250 GNLATEDLVYMLDGMGVETGVDLARLAQVGWRISDLLGRPPASRTSQA---------LRA
HMGCL_DROSOPHIL  267 GNAATEDVVYLLHGMGLDTGVNLDKLIQVGRYICTELGRTSESKVNRA----WKGPQARV
HMGCL_ARABIDOPS  409 GNVATEDVVYMLNGLGVHTNVDLGKLIAAGDFISKHLGRPNGSKAAVALNRRITADASKI
HMGCLL1_ZEBRAFI  284 GNVSTEDLLYMLHGLGIETGVDLLKVMEAGDFICKALNRKTNSKVSQA--------TRNN
HMGCLL1_XENOPUS  284 GNVATEDVIYMLNGLGIQTGVNLSKVMEVGHFICSALDRKTNSKVAQA--------SFQL
HMGCLL1_HUMAN    319 GNVATEDLIYMLNGLGLNTGVNLYKVMEAGDFICKAVNKTTNSKVAQA--------SFNA
HMGCLL1_MOUSE    289 GNVATEDLIYMLNGMGLNTGVDLYKVMEAGEFICKAVNKTTNSKVAQA--------SFNA
HMGCLL1_CHICKEN  287 GNVATEDVIYMLNGLGINTGVNLYTVMEAGNFICTALNKKTNSKVAQA--------SFNT
HMGCLL1_LIZARD   296 GNVATEDVIYMLNGLGINTGVNLYKVMEAGNFICTALNKNTNSKVAQA--------SFCG
HMGCL_XENOPUS    277 GNVATEDVVYMLHGLGIQTGIDLKKLTEAGAFICKALGKKSHSKVSQA--------ICKL
HMGCL_ZEBRAFISH  289 GNVATEDVVYMLHGLGIHTGVDLPRLLDAGSFICHSINRRTNSKVAQA--------SCKL
HMGCL_LAMPREY    259 GNVATEDVVYMLNGLGIQTGVDLQKLIEAGDFICKALNRRSGSKVSQA--------TSKL
HMGCL_HUMAN      274 GNLATEDLVYMLEGLGIHTGVNLQKLLEAGNFICQALNRKTSSKVAQA--------TCKL
HMGCL_MOUSE      274 GNLATEDLVYMLNGLGIHTGVNLQKLLEAGDFICQALNRKTSSKVAQA--------TCKL
HMGCL_CHICKEN    268 GNVATEDLVYMLNGLGIHTGVDLQKLMDTGTFICNALNRRTNSKVSQA--------ACRL
HMGCL_LIZARD     271 GNVATEDMVYMLHGLGIHTGVDLSKLLEAGAFICKALNRRTNSKVAQA--------SCKL

HMGCL_CELEGANS       ----
HMGCL_RHODOBACT  301 AQAG
HMGCL_DROSOPHIL  323 K---
HMGCL_ARABIDOPS  469 ----
HMGCLL1_ZEBRAFI  336 ----
HMGCLL1_XENOPUS  336 ----
HMGCLL1_HUMAN    371 ----
HMGCLL1_MOUSE    341 RLE-
HMGCLL1_CHICKEN  339 GAVK
HMGCLL1_LIZARD   348 ----
HMGCL_XENOPUS    329 ----
HMGCL_ZEBRAFISH  341 ----
HMGCL_LAMPREY    311 ----
HMGCL_HUMAN      326 ----
HMGCL_MOUSE      326 ----
HMGCL_CHICKEN    320 ----
HMGCL_LIZARD     323 ----
```

Supplementary Figure 1. Multiple sequence alignment of representative sequences from the HMGCL (mHL) and HMGCLL1 (er-cHL) family of proteins. Sequences were obtained from Uniprot (www.uniprot.org) and correspond to organisms: *Human, Mouse, Chicken, Anole lizard, Xenopus, Zebra fish, Sea lamprey, Drosophila, C. elegans, Arabidopsis* and *Rhodobacter*.
